# Association of Vitamin D Deficiency With Pulmonary Tuberculosis: A Systematic Review and Meta-Analysis

**DOI:** 10.7759/cureus.17883

**Published:** 2021-09-10

**Authors:** Sunam Kafle, Anjan Kumar Basnet, Kumar Karki, Manusha Thapa Magar, Shumneva Shrestha, Randhir S Yadav

**Affiliations:** 1 Internal Medicine, College of Medical Sciences, Bharatpur, NPL; 2 Internal Medicine, Health Foundation Nepal, Dang, NPL; 3 Internal Medicine, National Medical College, Birgunj, NPL; 4 Department of Pediatrics, Institute of Medicine, Tribhuvan University, Kathmandu, NPL

**Keywords:** tuberculosis, vitamin d deficiency, vitamin d, active pulmonary tuberculosis, vitamin d serum level

## Abstract

Pulmonary tuberculosis, caused by *Mycobacterium tuberculosis, *is a significant public health issue, especially in developing countries, affecting millions of people every year. Despite the development of many antitubercular antibiotics and increased awareness of preventive methods, it is still a major cause of mortality worldwide. Vitamin D, a micronutrient known to have a major role in bone and calcium metabolism, has also shown its immunomodulatory effects to suppress mycobacterial growth. We conducted a systematic review and meta-analysis of the available evidence to explore the association between vitamin D levels and tuberculosis. We performed a systematic search for articles from inception to May 2021 in multiple databases. We included 26 studies in our qualitative synthesis and 12 studies in meta-analysis or quantitative synthesis. In our meta-analysis, we used a random-effect model to calculate the odds ratio (OR) of vitamin D deficiency in tuberculosis patients compared to the healthy controls. On pooled analysis, we found that the odds of the participants having vitamin D deficiency was 3.23 times more in tuberculosis patients compared to the healthy group (OR=3.23, CI = 1.91-5.45, p<0.0001). Thus, we concluded that there is an association between low levels of vitamin D and tuberculosis infections. We suggest conducting long-term prospective cohort studies in tuberculosis endemic countries to better understand the causal relationship between vitamin D deficiency and tuberculosis.

## Introduction and background

Tuberculosis (TB), an infectious disease, is one of the top 10 causes of worldwide death, ranking above human immunodeficiency virus/acquired immunodeficiency syndrome (HIV/AIDS). In 2020, around 10 million people became ill, and 1.4 million died from TB [1-2]. The geographical distribution of TB in 2019 was 44% in the South-East Asia regions, 25% in Africa, 18% in Western Pacific, 8.2% in Eastern Mediterranean, 2.9% in the Americas, and 2.5% in Europe [2]. Immunomodulatory and anti-proliferative responses modulated by active 1,25-dihydroxy vitamin D were seen more than two decades ago. Its high doses were used to treat TB before discovering antitubercular drugs [3-4]. More understanding has been established in recent years regarding the effects of vitamin D in the pathophysiology and possible prevention of human disease, including TB [4].

The biological role of vitamin D in bone metabolism is well-established. However, the active metabolite of vitamin D, 1α,25-dihydroxy vitamin D3 (1,25D3) also has pleiotropic effects in the immune system [5]. 1,25 D3 upregulates human cathelicidin antimicrobial peptide and protein production from monocytes/macrophages infected with *Mycobacterium tuberculosis*, resulting in autophagy [6-8]. Vitamin D metabolites also upregulate nitric oxide synthase, which suppresses mycobacterial growth [9-10].

Over the years, more and more studies focusing on exploring the relationship between vitamin D deficiency and TB have been conducted [11-12]. However, existing attempts to relate these two things have been made with conflicting results caused by the different study populations, underlying comorbidities such as HIV and renal disease. Here, we analyzed the studies performed to find the association between vitamin D and TB. The studies we included were conducted in different countries and different age groups. We have tried to exclude any underlying comorbidities that may affect the level of baseline vitamin D.

The previous meta-analysis studies included cross-sectional and case-control studies, which assessed vitamin D status after the active TB disease diagnosis. The studies did not evaluate the efficacy of vitamin D supplementation in preventing TB [11-12]. Tuberculosis is now a global health problem, including in developed countries. The World Health Organization (WHO) End TB Strategy targets to decrease the incidence of tuberculosis by 80% by 2030 [2]. Around 1.7 billion people worldwide have latent tuberculosis infection, of which about 10% develop active tuberculosis in their lifetime [13-14]. To reach the 2030 target, effective measures to prevent acquiring the latent tuberculosis infection need to be addressed. Vitamin D supplementation is one of the interventions proposed to reduce the risk of latent TB infection in populations with prevalent deficiency [15-16]. To explore more about the vitamin D deficiency and tuberculosis association, we have systematically identified, examined, and pooled the community-and hospital-based studies that performed a comparative study of serum vitamin D in tuberculosis patients and healthy controls. We have also included a recent randomized control trial (RCT) assessing the role of vitamin D supplementation and the impact of pre-existing vitamin D levels on the risk of TB infection [15]. The inclusion of a large number of descriptional studies has made our study more robust. We believe the result will help give a clearer picture regarding the relationship between vitamin D and tuberculosis and help plan and execute future actions to control the tuberculosis infection in the world.

## Review

Methods

Data Source and Search Strategy

We followed Preferred Reporting Items for Systematic Reviews and Meta-analyses (PRISMA) guidelines to conduct our study [17]. Two reviewers performed rigorous literature searches on multiple databases, including PubMed, Embase, Scopus, and Cumulative Index of Nursing and Allied Health Literature (CINAHL). We searched for articles using medical subject headings (MeSH) and keywords combined with Boolean connectors, published from inception to May 21, 2021. We used both MeSH headings terms and regular keywords to conduct our literature search. We applied the 'human' filter while searching on PubMed and Embase. Likewise, the filter 'field of medicine' was used while searching on Scopus. The details of our search strategy on multiple databases are shown in Table 1.

**Table 1:** Search strategy of different databases

Database	Search Strategy	Records Retrieved
PubMed	((Tuberculosis[MeSH Terms]) OR (tuberculosis[Title/Abstract]) AND (humans[Filter])) AND (("vitamin d"[MeSH Terms] OR "ergocalciferols"[MeSH Terms] OR "vitamin d"[Title/Abstract] OR "cholecalciferol"[Title/Abstract] OR "ergocalciferol"[Title/Abstract]) AND (humans[Filter])) AND (humans[Filter])	800
Scopus	KEY ( ( vitamin AND d ) AND tuberculosis ) AND ( LIMIT-TO ( SUBJAREA , "MEDI" ) )	1295
Embase	('vitamin d':ab,ti OR ergocalciferol:ab,ti OR cholecalciferol:ab,ti) AND tuberculosis:ab,ti AND 'human'/de	1009
CINAHL	((Vitamin D) OR (ergocalciferol) OR (cholecalciferol)) AND (Tuberculosis)	198

Study Selection

**Figure 1:**
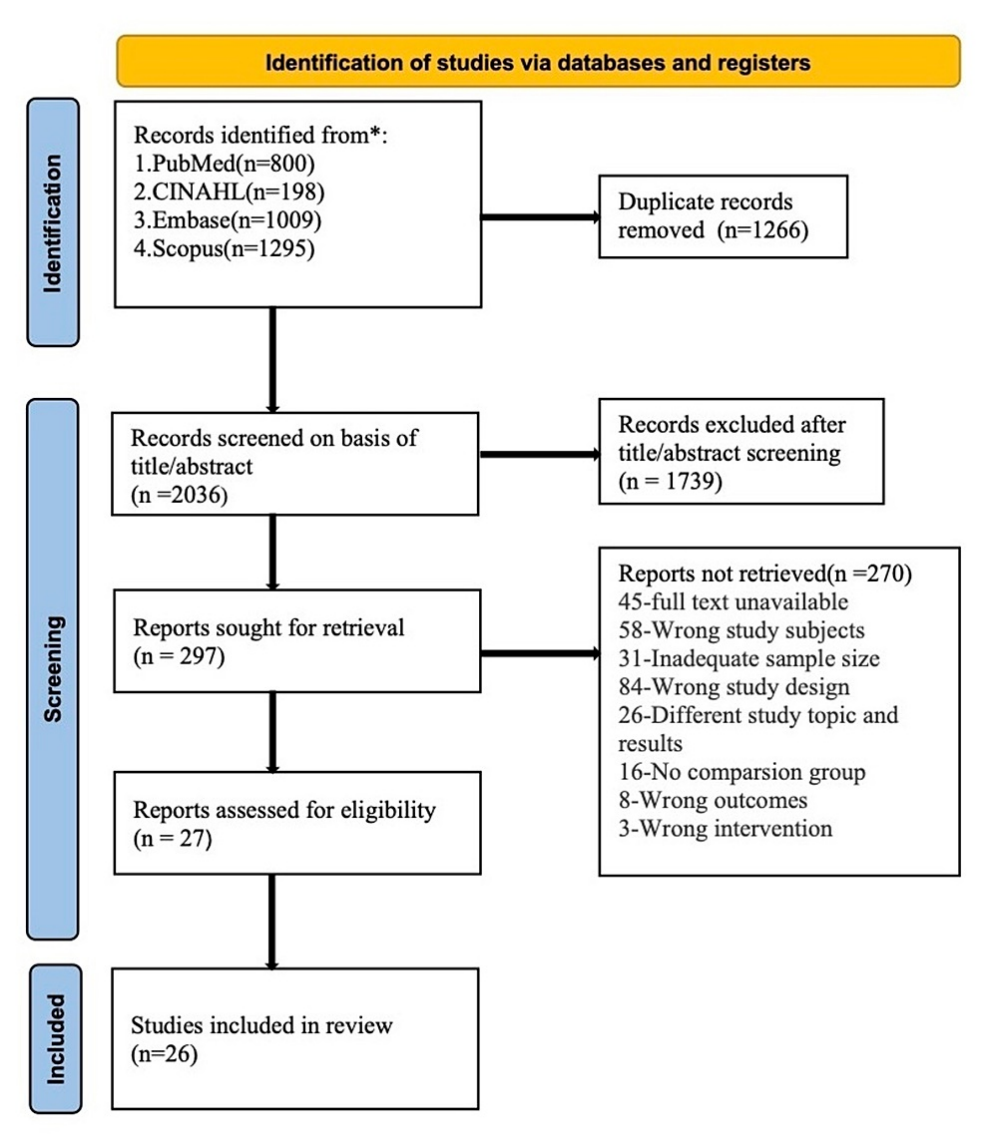
PRISMA flow diagram PRISMA: Preferred Reporting Items for Systematic Reviews and Meta-Analyses

We identified 3302 potentially relevant studies, including 800 from PubMed, 1009 from EMBASE, 1295 from SCOPUS, and 198 from CINAHL. The retrieved studies from the databases were imported to the Covidence review manager (CRM). We used CRM to remove the duplicate studies, and a total of 1266 duplicates were removed. Two reviewers independently carried out the whole screening process. We screened the titles and abstracts of 2036 articles and excluded 1739 studies that were not relevant to our research. Again, two reviewers retrieved and read the full text of the remaining 297 articles to check for the eligibility of the articles. The consensus of all six reviewers resolved disagreements during all stages of screening. Additionally, we searched the reference list of the relevant articles to identify any pertinent papers. Finally, we included 26 studies that met the requirement for our systematic review (Figure 1).

Inclusion and Exclusion Criteria

We included articles that have compared the level of serum vitamin D in patients with active tuberculosis and healthy controls of any age group. Given that the studies with small sample sizes may be statistically inconclusive, a study including a minimum number of 100 patients was taken for our review. The studies published in any language were included in our study. Case reports/series, review articles, meta-analysis, editorials, conference presentations, correspondence, commentary, poster presentation, and letters were not included. We excluded studies that had patients with latent tuberculosis, extra-pulmonary TB, and other comorbid conditions as their study participants. Animal studies and cellular models of studies were also excluded. Population Intervention Comparison Outcome (PICO) questions used for checking the eligibility of the studies are shown in Table 2. 

**Table 2:** Population Intervention Comparison Outcome (PICO) questions

P	Population/Problem/Patient	Patients diagnosed with active tuberculosis
I	Intervention	Vitamin D level
C	Comparison	Healthy patients without active tuberculosis
O	Outcome of interest	Mean or median vitamin D level or number of participants with vitamin D deficiency in both tuberculosis and control groups

Data Extraction

Two reviewers independently completed the data extraction from the finalized 26 studies. We included the following standardized forms: author, publication year, country, sample size, study population, mean age group, male to female ratio, study design, and outcome. All the relevant data from the included studies were extracted to an Excel sheet form (Microsoft Corporation, Redmond, WA). No automated tool was used for the data extraction process.

Study Risk of Bias Assessment 

We used the Joanna Briggs Institute (JBI) checklist for cross-sectional studies [18] and the JBI checklist for case-control studies [19]. We did a thorough quality assessment using the JBI checklist for 13 cross-sectional studies and 14 case-control studies.

For cross-sectional studies, we assigned score '1' to questions whose answers were 'yes' and score '0' to those whose answers were 'no' or 'unclear' or 'not applicable (N/A)'. After calculating the total score of all articles, we classified the studies with 6-8 as low risk, 3-5 as moderate risk, and 0-2 as high risk. Out of 13 cross-sectional studies, 12 cross-sectional studies were low-risk articles, and one was a moderate-risk article. Table 3 shows the quality appraisal for cross-sectional studies using the JBI checklist.

**Table 3:** JBI critical appraisal checklist for cross-sectional studies JBI: Joanna Briggs Institute

Study	Elsafi et al. (2020) [12]	Arfeen et al. (2020) [20]	Musharaf et al. (2020) [21]	Panda et al. (2019) [22]	Khan et al. (2018) [23]	Balcells et al. (2017) [24]	Ralph et al. (2017) [25]	Goyal et al. (2017) [26]	Workneh et al. (2017) [27]	Yuvaraj et al. (2016) [28]	Friis et al. (2013) [10]	Gray et al. (2012) [29]	Mastala et al. (2010) [30]
Were the criteria for inclusion in the sample clearly defined?	1	1	1	1	1	1	1	1	1	1	1	1	1
Were the study subjects and the setting described in detail?	1	1	1	1	1	1	1	1	1	1	1	1	1
Was the exposure measured in a valid and reliable way?	1	1	1	1	1	1	1	1	1	1	1	1	1
Were objective, standard criteria used for measurement of the condition?	1	1	1	1	1	1	1	1	1	1	1	1	1
Were confounding factors identified?	0	0	0	0	1	0	1	1	0	0	1	1	0
Were strategies to deal with confounding factors stated?	0	0	0	0	1	0	0	1	0	0	0	1	0
Were the outcomes measured in a valid and reliable way?	1	1	1	1	1	1	1	1	1	1	1	1	1
Was appropriate statistical analysis used?	1	1	0	1	1	1	1	1	1	1	1	1	1
Total score	6	6	5	6	8	6	7	8	6	6	7	8	6

Similarly, for case-control studies, we assigned 'one' to questions whose answers were 'yes' and 'zero' to the questions whose answers were 'no' or 'unclear' or 'not applicable (N/A)'. A score of 7-10 was considered low risk, 4-6 moderate risk, and 0-3 high risk. Out of 13 case-control studies, 11 studies were low-risk and two were moderate-risk articles. Table 4 shows the quality appraisal for case-control studies using the JBI critical appraisal checklist.

**Table 4:** JBI critical appraisal checklist for case-control studies JBI: Joanna Briggs Institute

Study	Jaimni et al. (2021) [11]	Wang et al. (2018) [31]	Zhang et al. (2018) [4]	Oh et al. (2017) [32]	Memon et al. (2016) [33]	Junaid et al. (2016) [34]	Gao et al. (2014) [35]	Kim et al (2008) [36]	Hong et al (2013) [37]	Iftikhar et al. (2013) [38]	Ho-Pham et al. (2010) [39]	Nielsen et al. (2010) [40]	Wejse et al. (2007) [41]
Were the groups comparable other than the presence of disease in cases or the absence of disease in controls?	1	1	1	1	1	0	1	1	1	1	0	1	0
Were cases and controls matched appropriately?	1	1	1	1	1	0	0	1	1	1	1	1	0
Were the same criteria used for the identification of cases and controls?	1	1	1	0	1	1	1	1	1	1	1	1	1
Was exposure measured in a standard, valid and reliable way?	1	1	1	1	1	1	1	1	1	1	1	1	1
Was exposure measured in the same way for cases and controls?	0	1	1	1	1	1	1	0	1	1	1	1	1
Were confounding factors identified?	1	0	1	1	1	1	0	1	0	1	1	1	1
Were strategies to deal with confounding factors stated?	0	1	0	0	0	1	0	0	0	0	0	0	0
Were outcomes assessed in a standard, valid and reliable way for cases and controls?	1	1	1	1	1	0	1	1	1	1	1	1	1
Was the exposure period of interest long enough to be meaningful?	1	0	0	1	0	1	1	1	1	0	1	0	0
Was appropriate statistical analysis used?	1	1	1	1	1	0	1	0	1	1	1	1	1
Total score	8	8	8	8	8	6	7	7	8	8	8	8	6

Results

Characteristics of the Included Studies

The included studies were published between 2007 and 2021. The included studies were published between 2007 and 2021. Thirteen included studies were case-control, and the other 13 were cross-sectional. Studies are included from various countries from multiple continents. A total of 8101 participants were included in our data synthesis; 4203 were people with TB and 3898 were healthy volunteers. All other studies have participants who are at least 14 years of age. Two of the studies from Pakistan have identical data and outcomes [20-21]. The details about the demographic features of the study participants, study design, and the year of publications are mentioned in Table 5.

**Table 5:** Characteristics of the studies included NA: not available; F: female, M: male, TB: tuberculosis

SN	Author	Year	Country		Sample size			Female/male ratio	Age range or mean/median age group (years)
					Total participants	TB cases	Healthy control		
1	Jaimni et al. [11]	2021	India	Case-control study	100	50	50	F=12, M=88	>18
2	Elsafi et al. [12]	2020	Sudan	Cross-sectional	201	101	100	F=72, M=129	21-40
3	Arfeen et al. [20]	2020	Pakistan	Cross-sectional study	140	70	70	F=52, M=88	20-70
4	Musharaf et al. [21]	2020	Pakistan	Cross-sectional study	140	70	70	F=52, M=88	20-70
5	Panda et al. [22]	2019	India	Cross-sectional study	150	80	70	F=28, M=122	18-60
6	Wang et al. [23]	2018	China	Case-control study	240	122	118	M=182, F=58	Cases: 50.83±20.04 Control: 51.97±12.49
7	Zhang et al. [4]	2018	China	Case-control study	187	128	59	F=46, M=141	NA
8	Khan et al. [23]	2018	India	Cross-sectional study	216	113	103	F=96, M=120	22.83-50.45
9	Balcells et al. [24]	2017	Chile	Cross-sectional study	262	92	170	-	NA
10	Ralph et al. [25]	2017	Malaysia	cross-sectional study	267	172	95	F=131, M=136	15-70
11	Goyal et al. [26]	2017	India	cross-sectional study	157	57	100	-	15-70
12	Workineh et al. [27]	2017	Ethiopia	Cross-sectional study	196	126	70	F=65, M=131	17.9-41.7
13	Oh et al. [23]	2017	Korea	Case-control study	289	152	137	F=98, M=191	18-80
14	Memon et al. [33]	2016	Pakistan	case-control study	209	112	97	F=84, M=125	NA
15	Junaid et al. [34]	2016	India	Case-control study	372	260	112	M=118, F=194	14-60
16	Yuvaraj et al. [28]	2016	India	Cross-sectional study	130	65	65	F=74, M=56	29.3-52.9
17	Gao et al. [35]	2014	China	Case-control study	227	74	153	F=100,M=127	31.2
25	Kim et al. [36]	2014	Korea	Case-control study	362	165	197	F=195, M=108	Case=46, Control=50
18	Hong et al. [37]	2013	South Korea	Case-control study	376	94	282	F=184, M=192	
19	Friis et al. [10]	2013	Tanzania	cross-sectional study	1570	1223	347	-	>15
20	Iftikhar et al. [38]	2013	Pakistan	Case-control study	360	105	255	F=155, M=205	39.78-57.46
21	Gray et al. [29]	2012	Australia	Cross-sectional study	247	11	236	F=150, M=178	0.5-17.5
22	Mastala et al. [30]	2010	Malawi	Cross-sectional study	318	161	157	M=165, F=153	NA
23	Ho-Pham LT et al. [39]	2010	Vietnam	Case-control study	385	166	219	F=159, M=232	NA
24	Nielsen et al. [40]	2010	Greenland	Case-control study	144	72	72	-	39
26	Wejse et al. [41]	2007	Guinea-Bissau	Case-control study	856	362	494	F=396, M=460	24.11-50.57
Total					8101	4203	3898		

Outcomes of the Included Studies 

Twenty-one studies [20-25,27,30-33,35-36,41] showed a significant association between the presence of active TB and vitamin D deficiency (VDD), i.e. VDD was found in active TB patients. Contrarily, in another study, high levels of vitamin D production were found in TB patients [29,39]. The mean of the majority of studies included in our study showed a higher than set criteria of VDD [30,38,41]. Further, few studies showed a lower mean value of vitamin D, which didn’t meet the set criteria of VDD [4,11,20-21,28,31,37]. Some other studies did not mention the reference level. The details of such values are mentioned in Table 6.

**Table 6:** Outcomes of the included studies SD: standard deviation; IQR: interquartile range; TB: tuberculosis; VDD: vitamin D deficiency

SN	Author	Mean ± SD of vitamin D level of the TB group	Mean ± SD of vitamin D level of the control group	Median and IQR of TB group	Median and IQR of the control group	Number of people with VDD in TB and control group	Criteria for VDD
1	Wejse et al. [41]	31.32 ± 9.04 ng/ml	34.12 ± 13.92 ng/ml	NA	NA	31/362 (TB) 65/494 (control)	<20ng/ml
2	Kim et al. 36]	13.5 ± 9.10 ng/mL	18.7 ± 8.33 ng/mL	NA	NA	73/165 (TB) 21/197(control)	<10ng/ml
3	Nielsen et al. [40]	NA	NA	NA	NA	Mild VDD: 9/72 (TB) 1/72 (control). No individuals had severe deficiency	<20ng/ml
4	Ho-Pham LT et al. [39]	Log[25(OH)D, ng/mL] Mean 3.40(0.24)	Log[25(OH)D, ng/mL] 3.39(0.18)	NA	NA	8/166(TB) 4/219 (control)	<20ng/ml
5	Mastala et al. [30]	23.88ng/ml	33.68+/-16n g/ml	NA	NA	26/157(TB) NA (control)	<20ng/ml
6	Gray et al. [29]	11.84 ng/mlL (95% CI: 9.2–15.2)	17 ng/ml (95% CI: 15.76 –18.36)	NA	NA	9/11 (TB) 132/236 (control)	<20ng/ml
7	Iftikhar et al. [38]	23.23 (±6.81) ng/ml	29.27 (±8.89) ng/ml	NA	NA	65/105 (TB) 85/255 (control)	
8	Friis et al. [10]	44.36 (14.28) ng/ml	33.76 (10.24) ng/ml	NA	NA	NA	-
9	Hong et al. [37]			9.86(7.19–14.15) ng/ml	16.03 (12.38–20.30) ng/ml	83/94 (TB) 209/282 (control)	<20ng/ml
10	Gao et al. [35]	365.9 (235.7) microgram/L	464.3(335.6) microgram/L]	NA	NA	NA	NA
11	Yuvaraj et al. [28]	15.4±6.8 ng/mL	17.5±5.7 ng/mL	NA	NA	NA	NA
12	Junaid et al. [34]	10.92 ng/ml	17.32 ng/ml	NA	NA	118/260 (TB) 33/112 (control)	<20ng/ml
13	Memon et al. [33]	27.1±9.7 ng/ml	36.8±8.1ng/ml	NA	NA	59/112 (TB) 19/97 (control)	<20ng/ml
14	Oh et al. [32]	NA	NA	10.6 (0.49-52.3) ng/mL	19.3 (6.2-60.5)ng/mL	124/152 (TB) 74/137 (control)	<20ng/ml
15	Workineh et al. [27]	12.04 ± 7.72 ng/ml	15.4 ± 8.36 ng/ml	NA	NA	105/126 (TB) 47/70 (control)	<20ng/ml
16	Goyal et al. [26]	13.9 ± 5.8 ng/ml	29.5 ± 6.5 ng/ml	NA	NA	NA	NA
17	Ralph et al. [25]	229.0 pmol/L, (95%CI 215.4-242.6)	153.9 pmol/L, (95% CI 138.4-169.4)	NA	NA	NA	NA
18	Balcells et al. [24]	NA	NA	11.7ng/ml	18.2ng/ml	71/92 (TB ) 92/170 (control)	<20 ng/ml
19	Khan et al. [23]	22.4±8.5 ng/dl	30.5±8.6 ng/dl	NA	NA	NA	NA
20	Zhang et al. [4]	10.42+/-5.06 ng/ml	21.97+/-6.90 ng/ml	NA	NA	122/128 (TB ) 25/59 (control)	<20 ng/ml
21	Wang et al. [31]	20.64+/-10.9 ng/ml	47+/-30.2ng/ml	NA	NA	NA	NA
22	Panda et al. [22]	-NA	NA	11.60 ± 5.1 ng/mL	21.50 ± 7.5 ng/mL	-NA	-NA
23	Musharaf et al. [21]	22.79±5.14 ng/ml	31.76±9.52 ng/ml	NA	NA	55/70 (TB group)	<25ng/ml
24	Arfeen et al. [20]	22.79±5.14 ng/ml	31.76±9.52 ng/ml	NA	NA	55/70 (TB ) 18/70 (control)	<25 ng/ml
25	Elsafi et al. [12]	10.68+/-0.64 ng/ml	46.92+/-1.28 ng/ml	NA	NA	NA	NA
26	Jaimni et al. [11]	NA	NA	19 (7.75, 27.25) ng/dl	25 (19.75, 32.00) ng/ml	27/50 (TB group) 13/50 (control)	<20 ng/ml

Meta-Analysis

**Figure 2:**
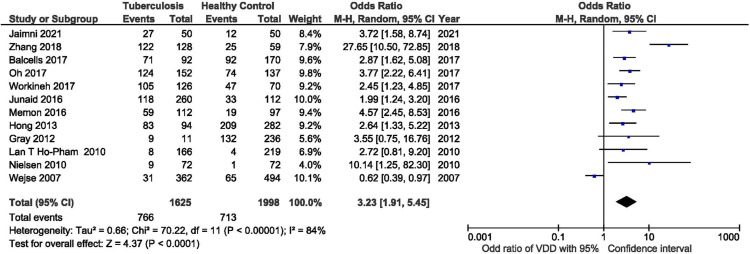
Meta-analysis showing the odds ratio of vitamin D deficiency in the tuberculosis group to the healthy control group [4,11,24,27,29,32-34,37,39-41]

We conducted a quantitative synthesis of only 12 studies from the total studies included in our systematic review (Figure 2). We used the Revman Manager version 5.4 (The Nordic Cochrane Centre, The Cochrane Collaboration, Copenhagen) to conduct our meta-analysis. In our study, we defined VDD as a concentration of vitamin D less than 20 ng/ml. We have included the studies that mentioned the discrete number of participants with vitamin D levels less than 20 ng/ml in both the TB and control groups. Therefore, only 12 studies were included in our meta-analysis. We did not include the studies that used a different cut-off for VDD to maintain the uniformity of the data. These results of the meta-analysis showed that the odds ratio (OR) of VDD in patients with TB to the healthy control is 3.23 with a confidence interval [1.91-5.45] (Figure 2). However, the result of our analysis showed that there is considerable heterogeneity in the included studies (I2=84%). The included studies vary in terms of sample size and age group of participants and geographical variation of the study population; therefore, we used the random-effects model to conduct our analysis.

Discussion

Most studies showed that lower vitamin D levels are more common among patients suffering from tuberculosis than in healthy volunteers. In 25 out of 26 included studies, the mean or median vitamin D concentration was higher in the healthy volunteers than in the TB patients. Also, the number of participants with VDD was found to be lower in the control group than in the TB group. Our review included the data from 8101 participants; 4203 were patients with tuberculosis infection and 3898 were healthy volunteers. Ralph et al. and Friis et al. were the only studies concluding the TB patients were likely to have a higher vitamin D level than healthy participants [10,25]. However, in the study by Ralph et al., the level of 1-25 hydroxyvitamin D was measured while in all other included studies, 25-hydroxy vitamin D was measured [25]. 1-25 hydroxyvitamin D is an active metabolite of vitamin D that is considered to be raised in granulomatous conditions such as sarcoidosis and pulmonary infections. According to Friis et al., the TB patients included in the study were under treatment and might have taken vitamin supplements [10]. These were the likely reasons for the contradictory finding of the higher vitamin D level in TB patients in these studies.

Our meta-analysis showed the TB patients were associated with lower vitamin D levels than the healthy controls. We calculated the odds ratio of VDD among tuberculosis patients to the healthy controls. The analysis's overall odds ratio (OR) was 3.23 with a confidence interval (CI) of 1.91-5.45 (Figure 2). This showed that the OR calculated was statistically significant, and there was a greater chance of people being vitamin D deficient in the tuberculosis group than in the control group. Our analysis included 1625 patients with tuberculosis and 1998 healthy controls. Out of the 12 studies included in our statistical analysis, the study by Wejse et al. was the only study with OR less than one. The OR of this study was 0.62 with a CI of 0.39-0.97 [41]. However, the mean vitamin D level in the tuberculosis patients was lower than that of the control group in this study [41]. We did not include the studies, which showed higher mean vitamin D levels in TB patients than in healthy volunteers because they had not mentioned the number of people with VDD [10,25]. Although our meta-analysis showed statistically significant increased odds of VDD in patients with TB compared to the healthy group, our analysis showed considerable heterogeneity (I2=84%) in the included studies. This heterogeneity could be due to variable sample size, non-uniformity in the ratio of people among TB and control groups, and larger CI in some studies. Therefore, the findings of this analysis should be applied with careful consideration.

The result of our meta-analysis is consistent with earlier meta-analyses [42-46]. However, in a meta-analysis by Zeng et al., a serum vitamin D level ≤ 25 nmol/L, 26-50 nmol/L, and 51-75 nmol/L was found to have significantly associated with an increased risk of tuberculosis, potential high tuberculosis risk, and no risk, respectively [43]. Moreover, in another meta-analysis done a few years later, Aibana et al. showed a dose-dependent association of Vitamin D and risk of TB where the risk was highest among HIV-positive individuals with severe VDD [44]. Our study did not examine the dose-dependent associations and comorbidities that may interfere with vitamin D levels. Like Huang et al. [42], our analysis found a significant association between TB and VDD, but it did not clarify if VDD was a risk factor for TB or its consequence. But their meta-analysis revealed that VDD was significantly associated with an increased risk of developing active TB in latent TB infected individuals or contacts of TB patients [42]. Since we did not include studies with latent TB infected individuals, our study cannot ascertain such associations. In this regard, vitamin D supplementation remains questionable for TB prevention and treatment [47]. Further studies may shed light on the clinical prospect of Vitamin D supplementation for TB prevention and treatment.

The strength of our study consists of the inclusion of all relevant studies, which justified reasonable quality to present a conclusive picture. Also, in our meta-analysis, we included studies with the same VDD cut-off to provide a decisive result. However, our study has certain limitations. There was a high heterogeneity noted because of the variations in the study designs. But a greater number of studies and participants points towards the robustness of the findings. Our study did not analyze the ethnic, environmental, and comorbidities that may have some role in the VDD. Our study supports the opinion of vitamin D supplementation in TB patients. It may also provide substantial evidence for future randomized clinical trials to investigate the role of vitamin D supplementation in TB prevention.

## Conclusions

We conducted a meta-analysis to identify the association between vitamin D level and tuberculosis infection. Our study has shown an association between low levels of vitamin D and tuberculosis. However, the studies included in our analysis have high heterogeneity, and they also do not clarify the causal relationship between the low vitamin D level and tuberculosis infection. Therefore, more prospective studies are required comparing the vitamin D-deficient group and healthy population and the development of tuberculosis infection in these groups to determine the causality.
